# High molecular weight hyaluronic acid: a two‐pronged protectant against infection of the urogenital tract?

**DOI:** 10.1002/cti2.1021

**Published:** 2018-06-07

**Authors:** Catherine A Mowbray, Syema Shams, Git Chung, Anna Stanton, Phillip Aldridge, Andrejus Suchenko, Robert S Pickard, Ased SM Ali, Judith Hall

**Affiliations:** ^1^ Institute of Cell and Molecular Biosciences Medical School Newcastle University Newcastle upon Tyne UK; ^2^ Institute of Cellular Medicine Medical School Newcastle University Newcastle upon Tyne UK; ^3^ Department of Urology Newcastle upon Tyne Hospitals NHS Trust Newcastle upon Tyne UK; ^4^ Department of Urology and Regional Spinal Injuries Unit Mid Yorkshire Hospitals NHS Trust Newcastle upon Tyne UK

**Keywords:** host defence peptides, hyaluronic acid, innate immunity, urinary tract infection, urothelial barrier

## Abstract

**Objectives:**

Recurrent urinary tract infections are associated with uropathogenic *Escherichia coli* (UPEC) ascending and infecting the urinary tract. Antibiotics provide only symptomatic relief, not prevent recurrence. Clinical evidence suggests that intravesical glycosaminoglycan therapy, such as hyaluronic acid (HA), helps reduce UTI recurrence. This has been investigated here using *in vitro* systems modelling the urogenital tract tissues.

**Methods:**

RT4 bladder cells were preconditioned with high molecular weight HA (> 1500 kDa) at 2 mg mL^−1^ and challenged with UPEC to analyse barrier protection and bacterial adherence. Untreated and HA‐preconditioned VK2 E6/E7 vaginal cells were challenged with *E. coli* flagellin (50 ng mL^−1^) to mimic bacterial challenge, and media analysed for lipocalin‐2, human β‐defensin 2 and interleukin‐8 by ELISA. Experiments were repeated after siRNA knockdown of Toll‐like receptors 2, 4 and 5, and CD44 to investigate signalling.

**Results:**

Microscopic analyses showed reduced bacterial adherence and urothelial disruption with HA, suggesting that HA functions as a barrier protecting the epithelium from bacterial infection. Cells treated with HA and flagellin simultaneously produced more of the host antimicrobial peptide LCN2 and pro‐inflammatory IL‐8 (*P *< 0.05) compared to the no HA/flagellin challenges. Increased gene expression of *DEFB4* (*P* < 0.05), but not the hBD2 peptide, was observed in the HA/flagellin‐challenged cells.

**Conclusion:**

These data suggest that exogenous HA has potential to protect the urogenital epithelia from UPEC infection via a two‐pronged approach that involves the physical enhancement of the epithelial barrier and augmentation of its innate immune response.

## Introduction

Urinary tract infection (UTI) is one of the most common bacterial infections with an estimated 150 million cases reported annually.^1^ Women are particularly affected, with up to one‐third of all women and more than half of all postmenopausal women suffering recurrence.^2^ Standard UTI treatment is antibiotic therapy, but for patients suffering from recurrent UTIs (rUTI), antibiotics provide only a short period of symptomatic relief before infection recurs. Moreover, the use of prophylactic antibiotics to treat rUTI is underpinning public health concerns about the consequences of antibiotic resistance^3^ and driving the need for alternative nonantibiotic prophylactic options to treat rUTIs.^4^

In females, UTIs are caused when bacteria originating in the gastrointestinal tract colonise the vagina and peri‐urethral area, and ascend to the bladder where they cause infection.^5^ Such organisms include *Staphylococcus, Proteus*,* Enterobacter*,* Klebsiella*,* Pseudomonas* and *Enterococcus* although the versatile pathogen *Escherichia coli,* known specifically as uropathogenic *E. coli* (UPEC), accounts for > 70% of all infections.^6^ Innate mechanisms are key in protecting the urinary tract from infection. These include physical factors such as the flushing action of urine which, together with its acid pH and ionic composition, defend the urogenital tract against bacterial colonisation and adherence to the urothelium. Additionally, Toll‐like receptors (TLRs) located in the lower urogenital tissues respond to microbe‐associated molecular patterns, specifically flagellin, and trigger the release of host defence peptides as well as inflammatory molecules that function to clear potential UTIs.^7^, ^8^ The importance of the TLRs in defence of the urinary tract (UT) is emphasised by studies where individuals carrying the TLR5_C1174T (R392STOP) and TLR2_G2285A (R753Q) SNP genotypes link to an increased risk of infection.^9^, ^10^, ^11^

Postmenopausal women suffer more frequently from UTIs,^2^ although the mechanisms controlling their increased susceptibility are unclear. In the vaginal tissues, oestrogen stimulates the production of glycogen, which is metabolised by the vaginal *Lactobacilli* populations to produce lactic acid. It is proposed that the lactic acid maintains an acid pH which helps to protect the vaginal tissues from colonisation by potential uropathogens including *E. coli*. Postmenopausal women are characterised by reduced oestrogen levels and those suffering rUTIs typically show a vaginal pH above 4.5,^12^ suggesting that the reduced oestrogen levels impact negatively on *Lactobacilli* growth, which adversely affects the vaginal microbiome.^13^, ^14^, ^15^ In support of a role for oestrogen in the innate defence of the urogenital tract, topical, but not oral oestrogen treatments have proven successful in reducing infections with these effects mediated through the vaginal commensal populations and the urogenital innate defences.^16^, ^17^, ^18^, ^19^ However, because of the side effects, the use of topical vaginal oestrogen is not always appropriate for all women^20^ and hence its therapeutic potential in treating rUTIs is limited.

Research for new therapeutic agents to help treat rUTIs has focussed on understanding the pathology of such infections, including knowledge of the virulence factors utilised by uropathogens to orchestrate an infection. UPEC are characterised by pili that carry FimH adhesion proteins that facilitate bacterial attachment through binding of mannosylated receptors on the urothelium. These structures are key to infection and have been targeted in the development of new therapeutics.^21^ However, initial strategies employing a vaccine approach and whole pili immunogens have proven ineffective, and other methodologies including the use of a FimC‐FimH complex, UPEC toxins and siderophores have reduced, but not totally inhibited, UPEC infection of the bladder.^22^, ^23^, ^24^ In contrast, agents called mannosides, which function as FimH antagonists and reduce bacterial attachment, show strong potential, with *in vivo* approaches involving animal models demonstrating the efficacy of a new class, the *C*‐mannosides.^25^ Recently, the FimH antagonist M4284 when administered orally to mice has been shown to reduce UPEC colonisation of the gut microbiota.^26^ If proven clinically, this approach will underpin an oral treatment available to all those suffering rUTIs that functions by reducing the faecal carriage of UPEC and hence the incidence of UTIs. An alternative approach to reduce UTI episodes in UTI‐prone patients is the deliberate inoculation of the bladder with a nonadhering *E. coli* strain, 83972, to establish asymptomatic bacteriuria.^27^ However, this procedure involves catheterisation and is invasive for the patient and, while protective, reduces rather than eliminates infections.

Other potential therapeutic agents include the glycosaminoglycans (GAGs), hyaluronic acid (HA) and chondroitin sulphate. Glycosaminoglycan instillations when compared clinically to placebo and antibiotics have been found to be linked to fewer episodes of UTI recurrence in women and longer periods between infections.^28^, ^29^, ^30^ Hence, these agents appear protective against UTIs. Again, however, clinical treatments involve an invasive catheterisation procedure. More acceptable therapeutically is a vaginal topical agent applied by the rUTI patient that functions as a barrier and prevents uropathogens from colonising the vagina, and ascending via the urethra to the bladder.^11^ To explore the potential of HA as a topical treatment for rUTI, this study used RT4 bladder and VK2E6/E7 vaginal cells to model the urogenital tract *in vitro*. These cells were preconditioned with HA and the effects of an UPEC challenge on the epithelial barrier and epithelial innate response examined using imaging and host peptide gene expression and protein analyses.

## Results

### Hyaluronic acid and the urothelial barrier

**Figure 1:**
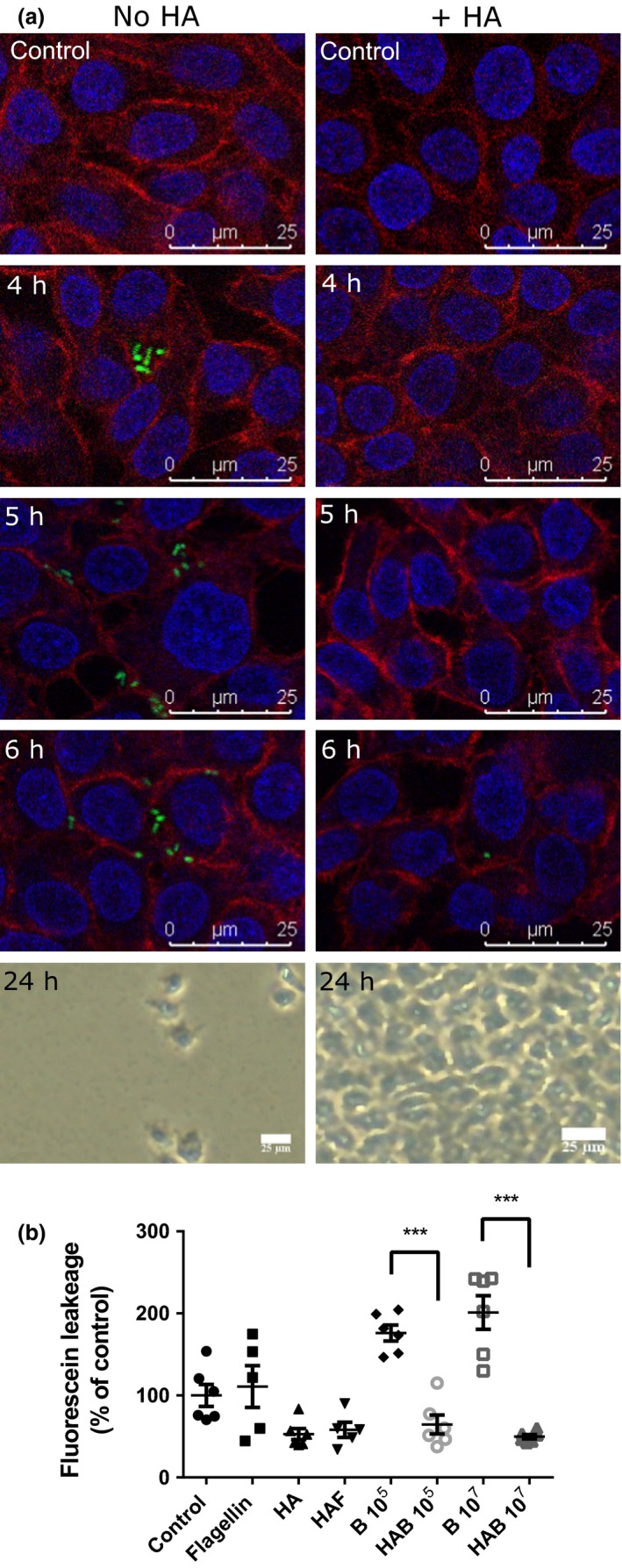
UPEC infection of urothelia. RT4 cells either alone or preconditioned with hyaluronic acid (HA; 2 mg mL^−1^) were exposed to GFP‐expressing UPEC (TPA4935J) for up to 24 h and microscopic images captured. Cells were fixed and stained with phalloidin (red: actin cytoskeleton) and DAPI (blue: nucleus) **(a)**. RT4 cells with or without hyaluronic acid (2 mg mL^−1^) were assessed for paracellular flux of fluorescein in presence of UPEC flagellin (50 ng mL^−1^) or 10^5^ bacteria over a 60‐min period **(b)**. Data are mean ± SEM (*N* = 3 experiments); ***P < 0.001. HAF, HA/Flagellin; B, Bacteria; HAB, HA/Bacteria.

Confluent urothelial cell monolayers cultured in medium containing either HMW (1500–1800 kDa) HA (2 mg mL^−1^) or no HA were challenged with a flagellated UPEC strain, TPA4935J, isolated from the urine of a rUTI patient and modified to express GFP. UPEC adherence to the unprotected cell monolayer (no HA) was detected within 4 h of infection, with the fluorescent bacteria locating specifically to the tight junctions of the RT4 cell monolayer and no visible adherence to other cell surfaces (Figure 1a). Increasing bacterial adherence was observed with incubation time, and by 24 h, the urothelial cell monolayer was severely disrupted. In contrast, bacterial adherence was not observed in the cells treated with HA until 6 h postinfection and the numbers of adhering bacterial colonies were limited (Figure 1a, Supplementary figure 1). At 24 h post‐UPEC infection, the monolayer of the HA‐treated cells remained intact, suggesting that the HA was functioning as a barrier and protecting the urothelium from bacterial adherence, and hence infection.

This barrier function was further supported by paracellular flux measurements in which confluent layers of bladder cells either preconditioned or not with HA (2 mg mL^−1^) were challenged with either UPEC or flagellin, a key UPEC virulence factor used to mimic infection. In the RT4 bladder cells preconditioned with HA, the passive leak of fluorescein (Figure 1b) and mannitol (Supplementary figure 2) was reduced (*P* < 0.01) compared to control. Moreover, flux was not affected when the cells were challenged with either UPEC or flagellin (F). However, in the absence of HA and presence of UPEC, cell flux was significantly increased compared to control (*P* < 0.05; Figure 1b). These data strongly suggest that the HA functions to tighten the epithelium and enhance the physical integrity of the epithelial monolayer.

### Hyaluronic acid and the urothelial innate response to UPEC flagellin

**Figure 2:**
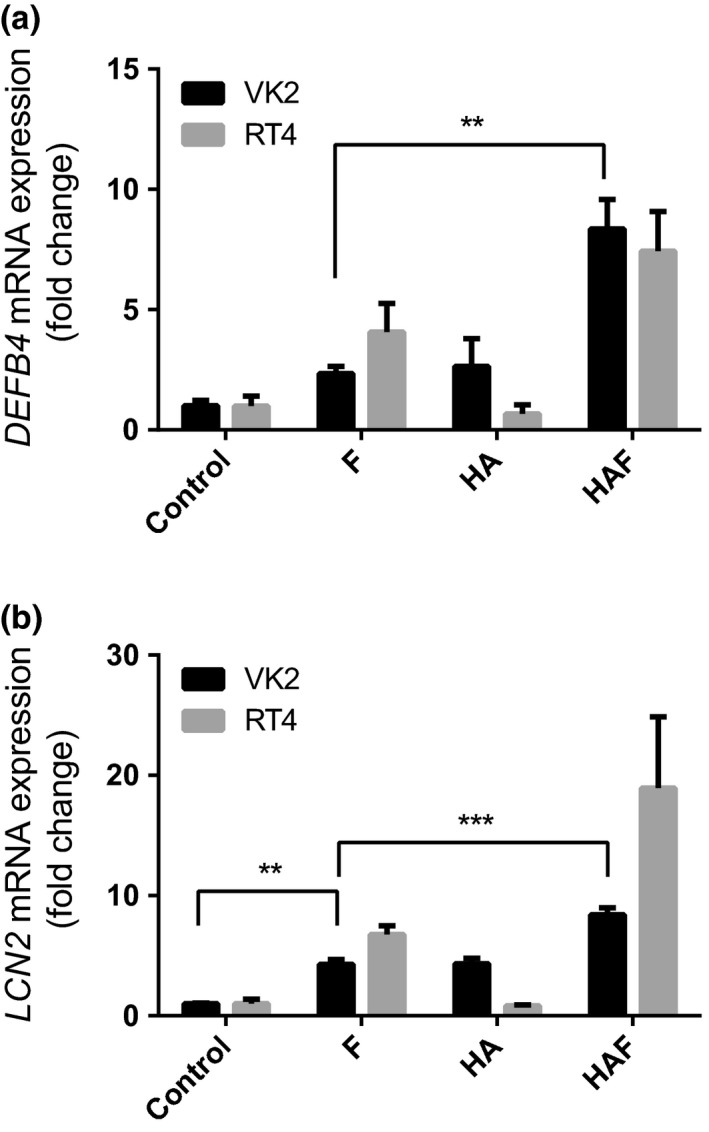
*DEFB4* and *LCN2* expression in cells preconditioned with hyaluronic acid. Hyaluronic acid 1500–1800 kDa (2 mg mL^−1^) was applied to VK2 E6/E7 and RT4 cells and either PBS or flagellin (50 ng mL^−1^) added, the latter to simulate an UPEC infection for up to 24 h. Gene expression of *DEFB4* **(a)** and *LCN2* **(b)** was measured by qPCR. Data are mean ± SEM (*N* = 3 experiments); **P < 0.01, ***P < 0.001. F, Flagellin; HA, Hyaluronic acid; HAF, HA/Flagellin.

The innate response is pivotal in protecting the urogenital tract from infection.^7^ Previous studies have shown that incubating vaginal cells *in vitro* with low molecular weight (LMW) HA (< 200 kDa) elicits an innate immune response characterised by the synthesis of host defence (HD) molecules, including the defensins 1–3 and lactoferrin.^31^ In contrast, treatment of vaginal VK2 E6/E7 and bladder RT4 cells for 24 h with HMW HA did not affect the expression of either the *DEFB4* or *LCN2* genes, encoding the host defence agents BD2 and LCN2 (NGAL; Figure 2a and b). However, challenging the HA‐preconditioned cells for 24 h with UPEC flagellin (50 ng mL^−1^) significantly enhanced gene expression (*P* < 0.01) compared to flagellin alone. These data indicated that preconditioning the urogenital cells with HMW HA augmented the innate immune response of these epithelia to flagellin, a key UPEC virulence factor.

If HA is to be considered for use as a vaginal topical agent then the mechanisms regulating the enhanced HA/flagellin signalling response require elucidation. TLRs 2 and 4 and CD44 each represent cell surface receptors of HA^32^ and the potential roles of these receptors in enhancing the innate response of the vaginal cells to flagellin, in the presence of HA, was explored using a gene knockdown approach (Supplementary figure 3). The effects of silencing the *TLR5* gene encoding TLR5, which mediates the host response to flagellin, were also examined.

**Figure 3:**
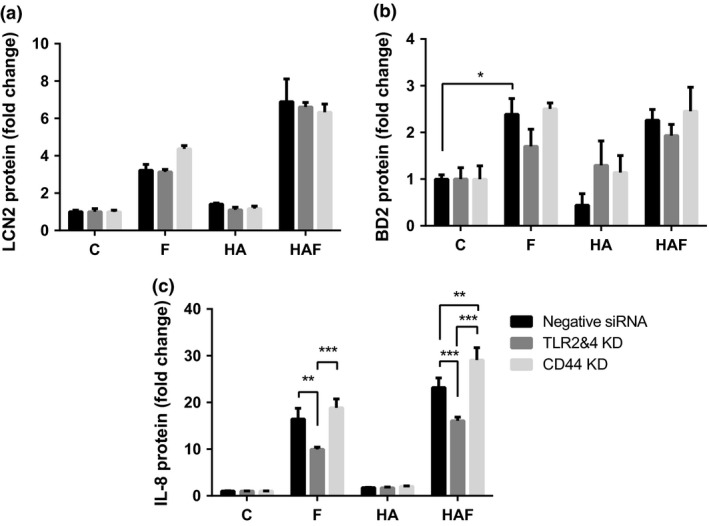
IL‐8, BD2 and LCN2 measurements following TLR2/4 and CD44 receptor gene knockdowns. VK2 cells were subjected to siRNA control knockdown (black bars), TLR2&4 together (dark grey bars) or CD44R knockdown (light grey bars). After 48 h, cells were incubated for 24 h plus/minus hyaluronic acid and challenged with flagellin (50 ng mL^−1^). Media postchallenge was collected, and LCN2 **(a)**, BD2 **(b)** and IL‐8 **(c)** concentrations were measured by ELISA, and data presented as fold change. Data are mean ± SEM (*N* = 2 experiments, *n* = 6 technical replicates); *P < 0.05, **P < 0.01, ***P < 0.001. C, Control; F, Flagellin; HA, Hyaluronic acid; HAF, HA/Flagellin.

Analyses of the VK2 E6/E7 vaginal cell media for the host protein LCN2 following the HA/flagellin challenge supported the gene expression data (Figure 2b), with significantly elevated concentrations of LCN protein (*P* < 0.01) compared to flagellin alone (> 2 fold change) observed (Figure 3a). These increases were not, however, affected following the TLR2/4/CD44 gene knockdowns. The BD2 protein concentrations in media collected from flagellin‐challenged cells also increased (*P* < 0.05) compared to control. However, the concentrations measured in the HA/flagellin‐challenged cells did not support augmentation of the innate response (Figure 3b) with comparable BD2 profiles observed in the media of the TLR2/4 and CD44 gene knockdown cells.

Measurement of the inflammatory protein marker, IL‐8, did not show significant protein concentration changes in the media in response to HA alone, suggesting that the HA treatment was not associated with inflammatory effects in the vaginal cells (Figure 3c). This observation was also supported by the lack of increase in *IL1b* gene expression (Supplementary figure 4). Significantly increased IL‐8 concentrations were observed when the cells were challenged with *E. coli* flagellin and these were also enhanced (*P* < 0.05) in the presence of HA. Augmentation of the IL‐8 protein response was similarly observed in all the knockdown experiments although these data suggested the involvement of TLR2/4.

**Figure 4:**
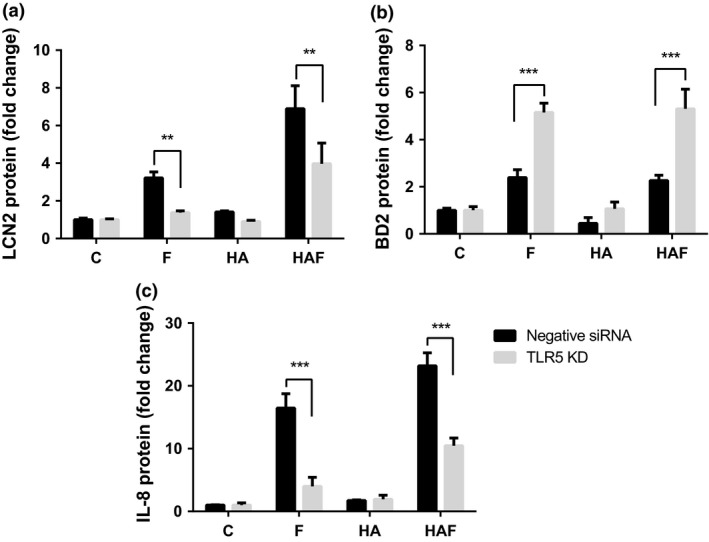
IL‐8, BD2 and LCN2 measurements following TLR5 receptor gene knockdown. VK2 cells were subjected to siRNA control knockdown (black bars) or TLR5R (grey bars) knockdown. After 48 h, cells were incubated for 24 h plus/minus hyaluronic acid and challenged with flagellin (50 ng mL^−1^). Media postchallenge were collected, and LCN2 **(a)**, BD2 **(b)** and IL‐8 **(c)** concentrations were measured by ELISA and data presented as fold change. Data are mean ± SEM (*N* = 2 experiments, *n* = 6 technical replicates); **P < 0.01, ***P < 0.001. C, Control; F, Flagellin; HA, Hyaluronic acid; HAF, HA/Flagellin.

Following TLR5 knockdown, reduced concentrations of LCN and IL8 were detected in the media following flagellin challenge (Figure 4a and c), but augmentation of the response was still observed in the challenged cells pretreated with HA compared to flagellin alone. In contrast, flagellin treatment of the *TLR5* gene knockdown cells was associated with an increased BD2 response compared to the control siRNA cells. HA/flagellin treatment of such cells followed a similar pattern, but the presence of HA was not associated with an augmented response (Figure 4b).

### Hyaluronic acid and NFκB signalling

**Figure 5:**
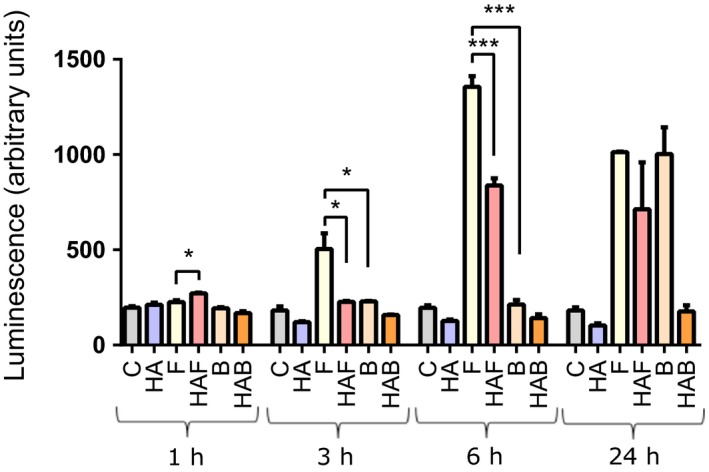
NFkB signalling in cells preconditioned with hyaluronic acid and challenged with UPEC or flagellin. HA and RT4 cells expressing an NFkB luciferase reporter gene were challenged with HA (2 mg mL^−1^), F (50 ng mL^−1^) or heat‐killed UPEC (10^4^ per well) for up to 24 h. Cells were subsequently lysed, luciferase substrate added and the resulting luminescence recorded. Data are mean ± SEM (*N* = 3 experiments, *P < 0.05, ***P < 0.001). C, Control; HA, Hyaluronic acid; F, Flagellin; HAF, HA/Flagellin; B, Bacteria; HAB, HA/Bacteria.

The transcription factor NFκB is a key signalling molecule functioning in the innate response of the urogenital cells to flagellin^11^ and the response to HA.^33^ The signalling responses of VK2 E6/E7 and RT4 cells are comparable in their NFκB response to flagellin^11^; therefore, to further explore the signalling mechanisms underpinning the HA/flagellin effects, urothelial cells stably engineered to contain an NFκB reporter were used. These cells were incubated with flagellin (F), HMWT HA, HA/flagellin (HAF), heat‐killed UPEC (TPA4935) (B) and HA/B, and NFκB signalling measured from one to 24 h following challenge (Figure 5). HMWT HA treatment alone did not induce NFκB activity up to 24 h, supporting its noninflammatory properties. By 3 h of flagellin treatment NFκB signalling was evident, although no response was observed in the flagellin‐treated cells preconditioned with HA, supporting it functioning as a protective barrier. At 6 h, NFκB activity was detected in the HA/flagellin‐treated cells, but at a reduced level (*P* < 0.05) compared to the cells treated with flagellin alone. The NFκB signalling response to the HA/B‐challenged cells remained comparable to control levels for up to 24 h. These data were consistent with the HA forming a protective layer and helping to protect the epithelium from whole bacteria and flagellin proteins.

## Discussion

The glycosaminoglycan HA is found naturally throughout the body as a major constituent of the extracellular matrix,^34^ and functions in healthy tissues to regulate cell growth, differentiation and to inhibit inflammation.^32^ Clinically, bladder instillations containing HA have been shown to reduce UTI episodes and pain^30^, ^35^, ^36^ with *in vivo* studies suggesting that HA treatment is associated with reduced bacterial growth.^37^ However, these are only used in cases of extremely unresponsive rUTIs.^20^ A recent clinical trial has reported the effects of HA combined with *Propionibacterium acnes* as a potential treatment to combat vaginal infections, functioning presumably by enhancing the immune system in the vaginal/vulval tissues.^38^ While this therapy did reduce the symptoms of bacterial vaginosis, the study lacked a control arm treated with HA alone, hence the actual contribution of HA cannot be ascertained. Although the literature reporting the vaginal use of HA as a topical agent to treat UTI incidence *in vivo,* either through clinical human trials or animal studies, is sparse the evidence to date does support a role for HA in protecting the urogenital tract from infection.

To further explore the mechanisms by which HA helps protect from UTI an *in vitro* approach exploiting RT4 and VK2 E6/E7 cells, modelling the urogenital tract tissues, was used. Cells challenged with UPEC resulted in bacteria being localised to the tight junctions of the urothelium, which over 24 h resulted in a weakened, disrupted epithelial barrier concomitant with UTI pathology. These data endorsed previous observations,^39^, ^40^, ^41^ and supported an UPEC infection mechanism involving manipulation of the paracellular pathway. Tight junction investigation in primary urothelial cells has shown that claudin 3, ZO‐1 and ZO‐1α^+^ are important for the correct functionality of tight junctions in urothelia,^42^, ^43^ with the loss of ZO‐1 associated with UPEC infection of bladder epithelia^39^ and a potential UPEC target.

Preconditioning the cells with HA reduced bacterial attachment and epithelial disruption, which supported the glycosaminoglycan as protecting the epithelial barrier physically. Although the cell line model does not take into account urinary flushing and hence potential loss of the HA layer, it does demonstrate the positive effects of using HA to help protect the urogenital tract from infection. Clinically, we suggest using HA as a topical vaginal treatment rather than a bladder treatment, hence we predict that any flushing effects would be reduced. One method by which UPEC has been reported to infect the murine urinary tract is through attaching to CD44 receptors^44^ that also bind HA.^45^ Although receptor masking by HMW HA may have functioned in reducing UPEC infection *in vivo*, specific localisation of UPEC to the epithelial tight junctions was less supportive of such a mechanism operating in the human urothelial cell model.

High molecular weight hyaluronic acid has been shown to protect against inflammation, and the mechanism is suggested to involve cross‐linking of the glycosaminoglycan with transmembrane CD44 receptors.^32^ The importance of the cell adhesion receptor molecule CD44 in this process has been further demonstrated *in vivo* using CD44^−/−^ mice, with the accumulation of LMW HA associated with and responsible for an exaggerated inflammatory response.^46^, ^47^ Consistent with the reported properties of HMW HA, its application to urothelial cells was not associated with inflammation as measured by either NFκB signalling or immune effector synthesis. Interestingly, however, the presence of exogenous HMW HA enhanced the innate response of the urogenital cells to *E. coli* flagellin, a virulence factor used *in vitro* to mimic an UPEC infection. Bacterial infection and cell injury are associated with hyaluronidase activity^48^ and the accumulation of LMW material. Moreover, the inappropriate clearance and persistence of LMW HA links to multiple inflammatory pathologies including arthritis, lung fibrosis^49^ and potentially interstitial cystitis.^50^ While the lack of innate responses to the HMW material alone suggested no contamination with LMW material, it cannot be excluded the endogenous production of LMW HA during the challenge period was responsible for the enhanced effector responses. LMW HA has been reported to function through TLRs 2 and 4, but independently of CD44, to stimulate the synthesis of host defence effectors including BD2 and SLPI in vaginal cells.^31^ Surprisingly, however, in view of its pro‐inflammatory properties,^32^ these responses observed using the VK2E6/E7 cell model were reported to be independent of pro‐inflammatory cytokine production.

The actual signalling mechanisms regulating the augmented urothelial innate response to HA/flagellin in our *in vitro* challenges remain uncertain. Our data, as predicted, supported a role for TLR5 but it did not strongly support either TLR2/4 or CD44 involvement. It cannot be excluded, however, that these data were prejudiced by the signalling activities of receptors persisting following incomplete gene knockdown. HMW HA treatment is not only associated with receptor binding but also receptor clustering that functions, potentially, to stabilise cell membrane transporters and/or receptors.^45^ Hence, if activated urothelial TLR5 and CD44 receptors cluster similarly in response to flagellin and HA, then the outcome supports signal amplification through either better retention of their respective ligands or increased rebinding. While the mechanisms underpinning signal amplification remain obscure, cooperation between receptors either physically or through shared intracellular signalling molecules cannot be excluded. The potential roles of other HA binding molecules such as the hyaladherins, RHAMM, LYVE‐1 and HARE^32^ also remain to be explored.

In contrast to LCN2 and IL‐8, it was surprising that no enhancement of the BD2 peptide response was observed in the presence of either flagellin or HA/flagellin, an observation that did not marry with the *DEFB4* expression data. BD2 peptide molecules are positively charged and have been reported to bind to negatively charged GAGs.^51^ It is therefore possible that precipitation of the BD2‐GAG complexes on the epithelial cell surface masked the *in vitro* increase in BD2 concentrations in response to flagellin. However, these data were further complicated by the enhanced BD2 responses following *TLR5* gene knockdown. Such observations may have reflected the responses of the cells to reduced TLR5 numbers including the recycling of endocytosed TLR5 receptors to the cell membrane, the unregulated release of BD2 from intracellular cell granules or other negative feedback mechanisms functioning to increase the synthesis and storage of BD2. As BD2 is a key effector in defending the urogenital tract^11^ and the enhanced responses appeared independent of NFκB signalling, further investigation of this response is required.

Overall, these *in vitro* data provide evidence for HMW HA functioning not only as a mechanistic barrier protecting the urothelium from UPEC infection, but also boosting the innate response of the epithelium in the presence of flagella, a key UPEC virulence factor. These two protective mechanisms, while independent, support previous *in vivo* observations reported using rodents^37^, ^52^ as well as complementing each other to limit bacterial growth. They also help to explain why HA instillations, clinically, are successful in reducing UTI episodes.^30^ These observations also suggest that therapeutic topical HA has potential as an alternative or co‐treatment to antibiotics in the management of recurrent UTIs. However, focussed clinical trials are required to underpin its therapeutic use as a topical agent.

## Methods

### Cell culture

Vaginal and bladder epithelia were modelled using VK‐2 E6/E7 (ATCC‐CRL2616) and RT‐4 (ATCC‐HTB‐2) cells respectively. VK2 cells, cultured in Keratinocyte‐SFM (ThermoFisher) with recommended supplements, were seeded into 12‐well plates (2 × 10^5^ cells per well). RT4 cells, cultured in RPMI with L‐glutamine (2 mm), HEPES (2.5%) and 10% FBS (Sigma), were seeded into 6‐well plates (5 × 10^5^ cells per well), or 12‐well transwells (1.2 × 10^5^ cells per well). RT4 with NFkB luciferase reporter plasmid^53^ were grown in RT4 media with G418 (0.5 mg mL^−1^) and seeded into 96‐well plates (5 × 10^4^ cells per well).

### Mimicking bacterial infection

Confluent vaginal or bladder cells were incubated with HA MW 1500–1800 kDa (Sigma; 0.2%, 2 mg mL^−1^) and UPEC flagellin (F) (50 ng mL^−1^)^11^ alone or together for up to 24 h. Media was collected for ELISA analyses and cells lysed for RNA extraction and qPCR.

### Epithelial flux

RT4 cells were grown on transwells for 14 days, followed by 1‐h incubation with HA (2 mg mL^−1^) and 6‐h challenge with either clinical UPEC isolate, 3412, (10^5^–10^7^ cells per mL) or flagellin (50 ng mL^−1^). To test whether HA protected the epithelial barrier, ^14^C‐mannitol (10 μm) or fluorescein (100 μg mL^−1^) was added to the apical compartment and media sampled from the basolateral compartment at time periods up to 1 h. Radiolabelled mannitol was detected using a scintillation counter and fluorescence measured using a FluoStar Omega microplate reader (BMG Labtech, Ortenberg, Germany).

### Luciferase assay

RT4 cells stably transfected with NFĸB luciferase reporter plasmid^53^ were challenged for up to 24 h with either flagellin (50 ng mL^−1^) or heat‐killed UPEC (10^4^ per well). Cells were lysed overnight at −80°C with reporter lysis buffer (Promega) and luciferase activity measured using a luciferase reporter assay kit (Promega). Luminescence was measured using the FLuostar Omega plate reader.

### Staining and imaging of cells

RT4 cells were seeded onto 8‐well chamber slides (Corning) at 3 × 10^4^ cells per well and grown to confluence. Cells either preconditioned or not with HA (2 mg mL^−1^) were challenged for up to 24 h with TPA4935J, a clinical UPEC isolate transformed with pEGFp‐C3 (10^2^ per well). After washing with PBS, fixing in 4% PFA the cells were stained for 2 h with CytoPainter Phalloidin‐iFluor 594 reagent (Abcam) diluted 1:1000, and coverslips mounted using Vectasheild with DAPI (Vector Laboratories). Images were captured using a Leica SP8 confocal microscope (fluorescence) or an EVOS XL Core Cell Imaging System (Thermo Fisher; light microscope images). Image analysis was conducted using LAS X (Leica) and Image J software.

### siRNA Knockdowns

Transfection of VK2 cells with siRNA (ThermoFisher) was performed using Viromer Green (Lipocalyx) at 65% confluence. siRNAs used were as follows: s168 (TLR2), s14196 (TLR4), s2681 (CD44), s14199 (TLR5), AM4611 (negative siRNA 1). Transfection was performed at ×0.5 reaction volume (single knockdown) or ×0.25 reaction volume (double knockdown) following the manufacturer's protocol for 48 h, followed by challenge with HA and/or flagellin as previously described for 24 h.

### RNA extraction and qPCR

RNA extraction used the SV Total RNA Isolation Kit (Promega), and nucleic acid was quantified by nanodrop. Reverse transcription of RNA (400 ng) used random hexamers (Roche), MMLV Reverse Transcriptase and RNase Inhibitor (Promega). mRNA expression was quantified by qPCR using SYBR Green (Roche) with 0.5 μm primers on a LightCycler 480 (Roche). Appropriate reference genes, *GAPDH* and *ATP5b* for VK2, *GAPDH* for RT4, were determined using GeNorm^54^ (Primer Design). Primer sequences of target genes can be found in Supplementary table 1. PCR products were verified by sequencing and controls were included in each plate, enabling verification of single target amplification using melt curve analysis. Data were normalised to the appropriate reference genes.

### ELISA

ELISA kits to measure protein levels of BD2 (Leinco Technologies), lipocalin‐2 (LCN2) and interleukin‐8 (IL‐8; both R&D Systems) in media harvested from cell challenges were used as per manufacturer's instructions. Absorbance was measured at 540 and 450 nm using a FLUOstar Omega (BMG Labtech, Germany) plate reader, with a standard curve and negative control on each plate.

### Statistical analyses

Data are presented as mean ± SEM, and statistical analyses were performed using the Prism 6 Software package (GraphPad Software Inc, La Jolla, California, USA). For analyses of data involving more than two groups, a one‐way analysis of variance followed by a Bonferroni post‐test at a significance level of *P* < 0.05 was used.
