# Supplementary figures and images for: High molecular weight hyaluronic acid: a two‐pronged protectant against infection of the urogenital tract?
Source: Clin Transl Immunology. 2018 Jun 7;7(6):e1021. doi: 10.1002/cti2.1021 (PMC5993165; doi:10.1002/cti2.1021)

## Slide 1
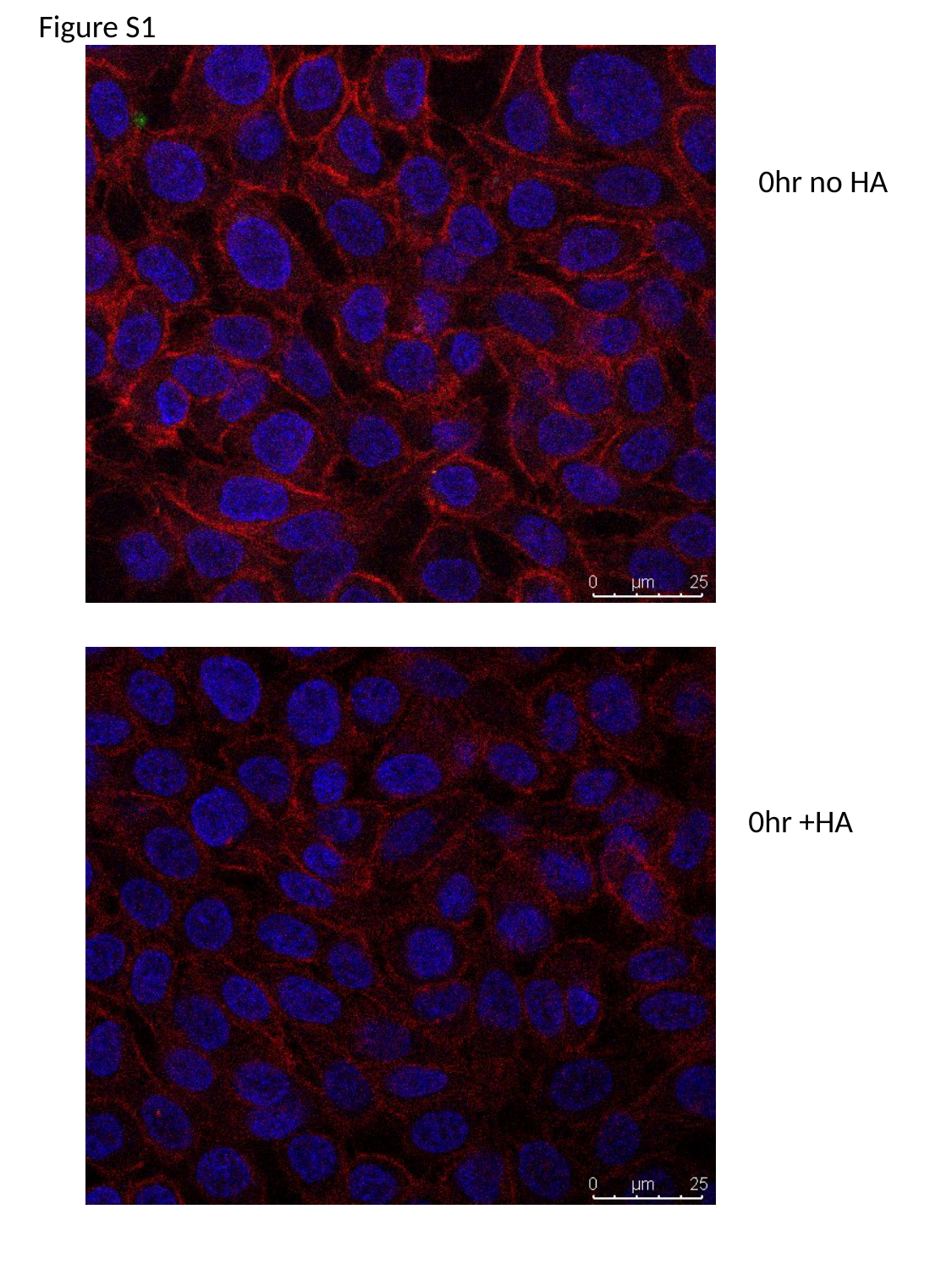

Figure S1
0hr no HA
0hr +HA

## Slide 2
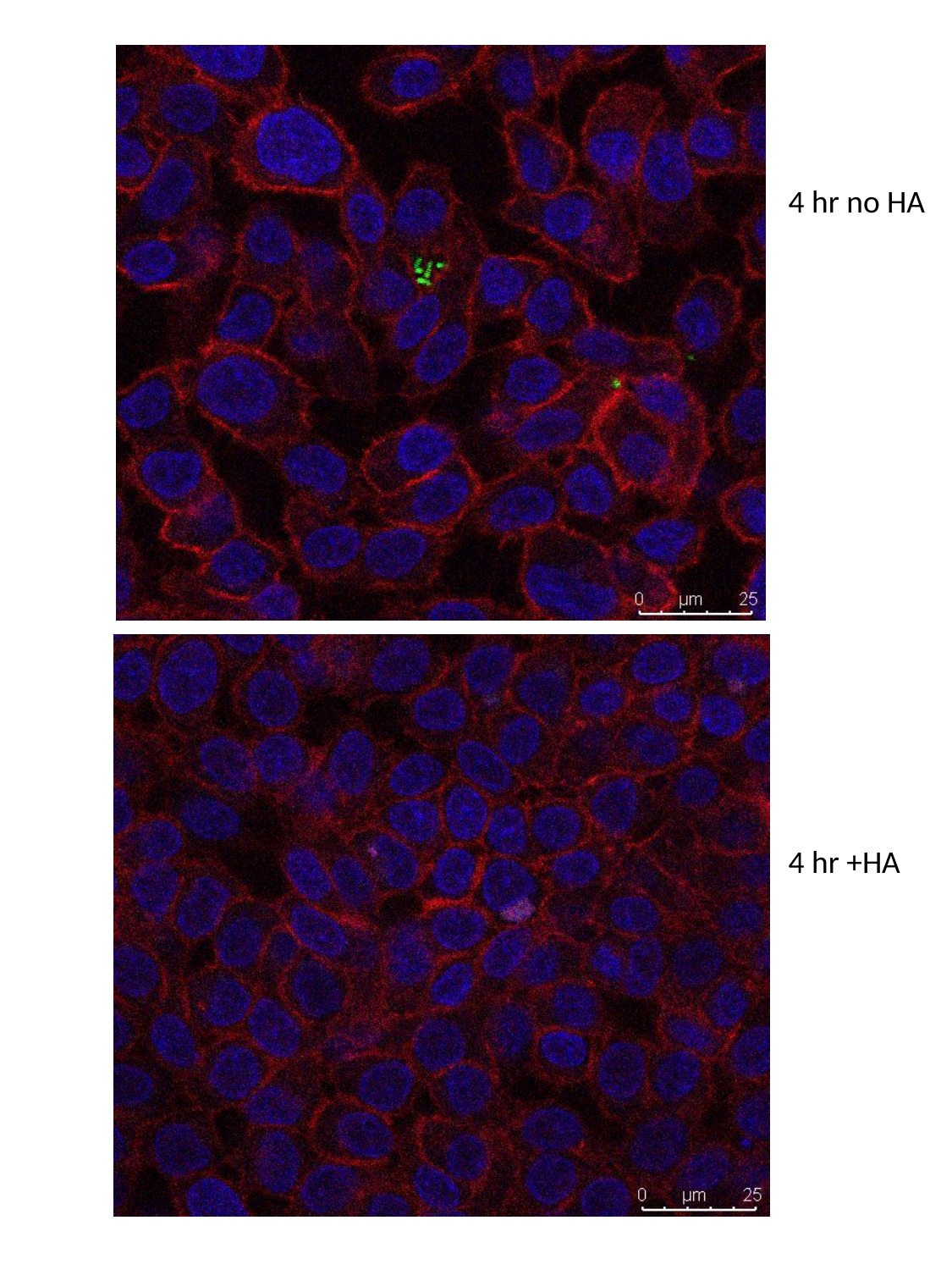

4 hr no HA
4 hr +HA

## Slide 3
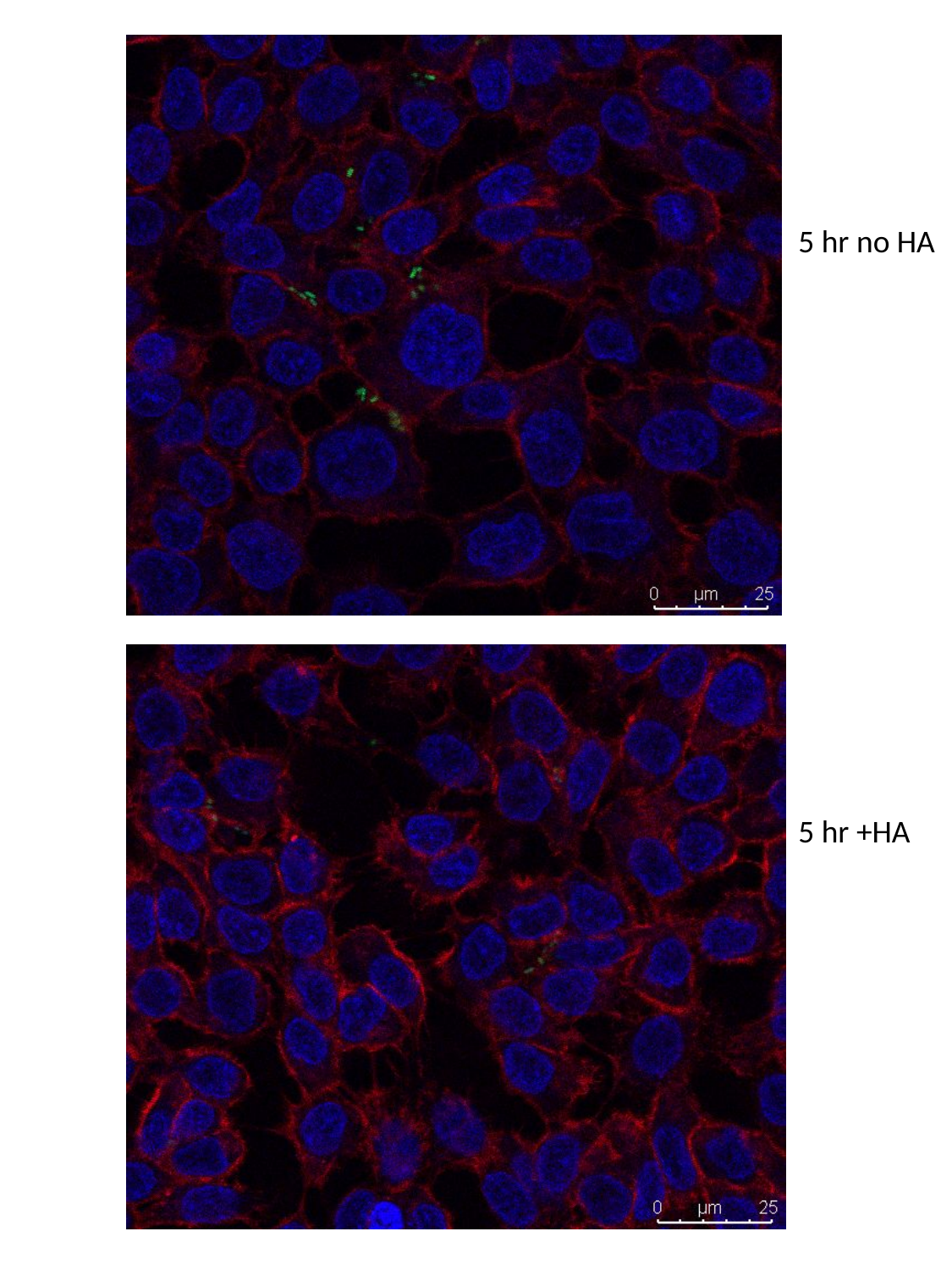

5 hr no HA
5 hr +HA

## Slide 4
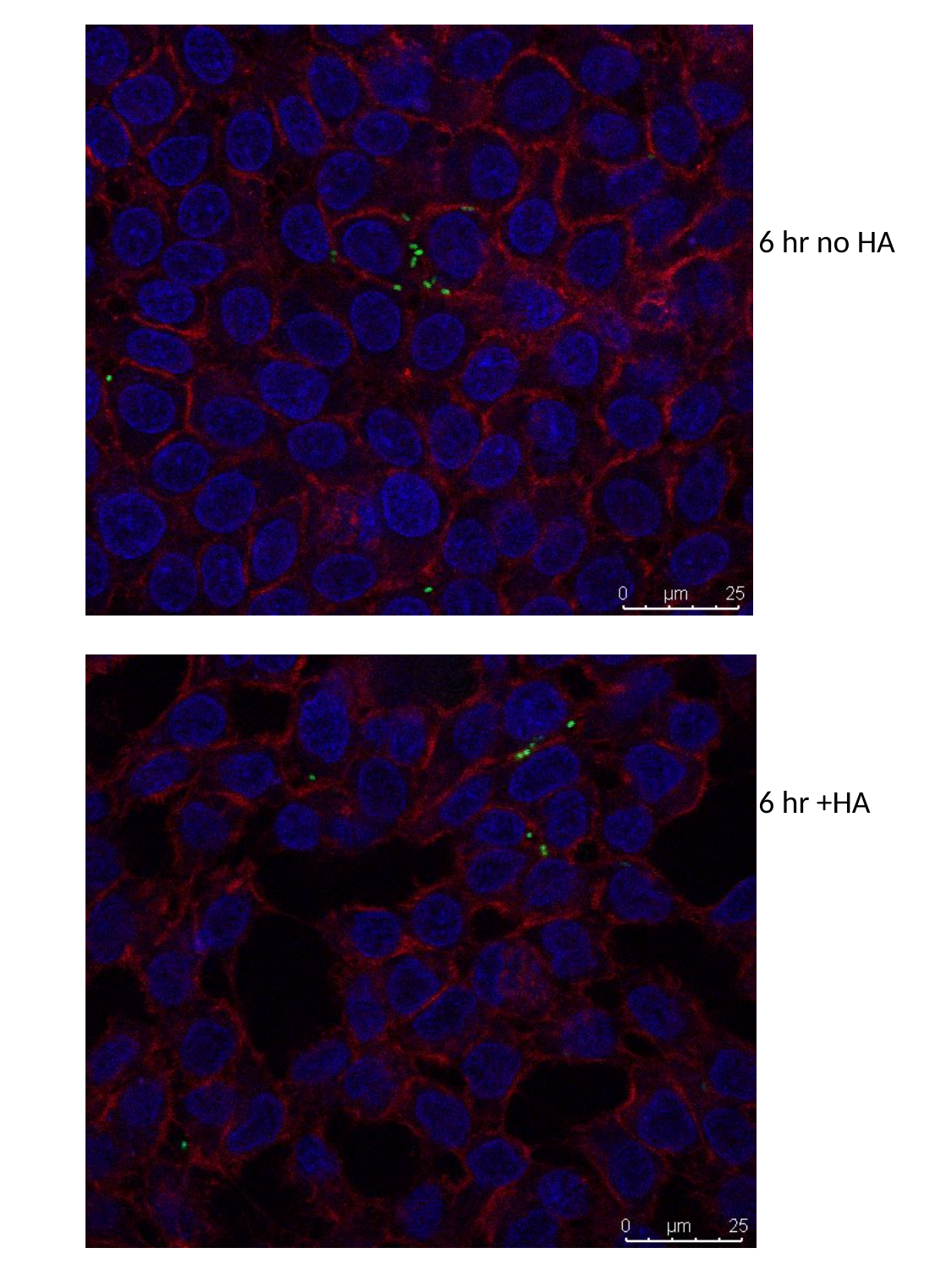

6 hr no HA
6 hr +HA

Supplement: Supplementary file 1 [file CTI2-7-e1021-s001.pptx]

Percentage of mannitol  
A-B paracellular flux

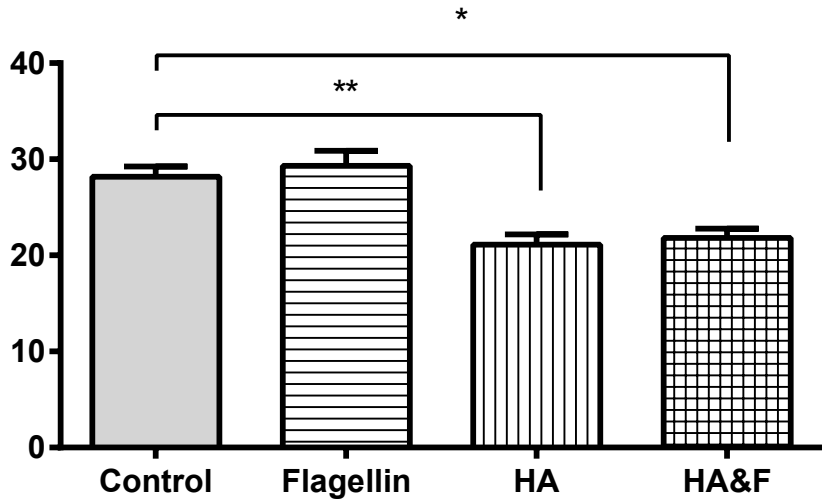

Supplement: Supplementary file 2 [file CTI2-7-e1021-s002.pdf]

# Supplementary Figure 3

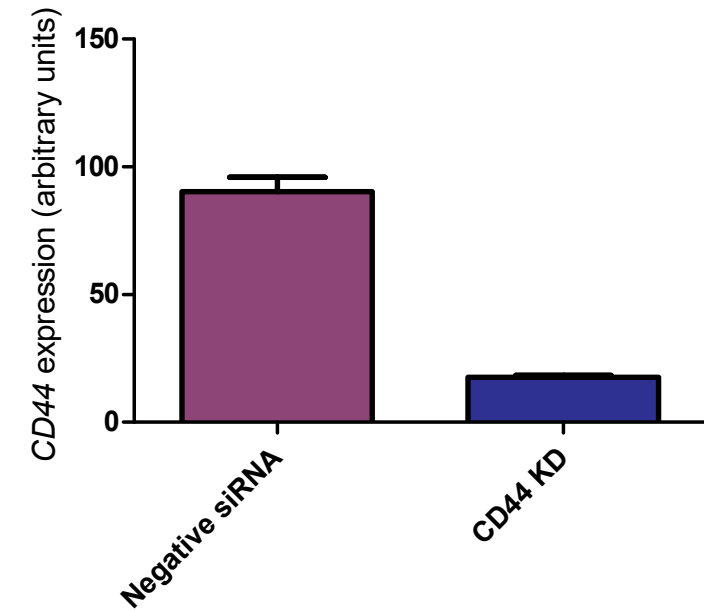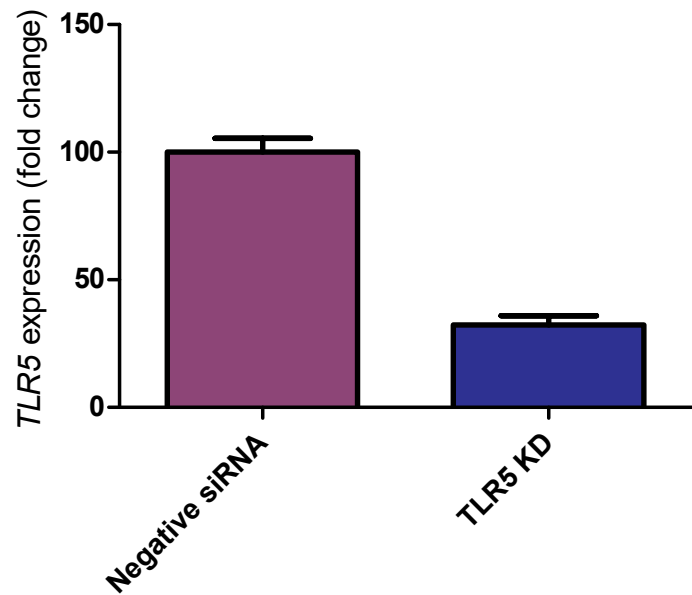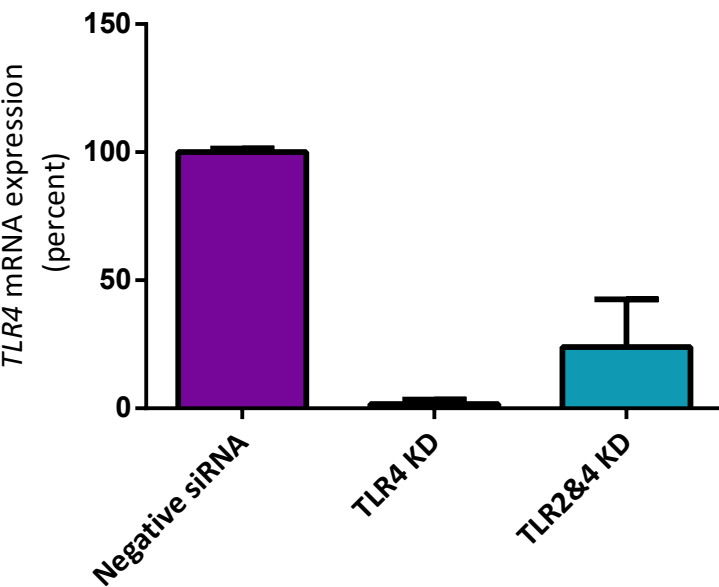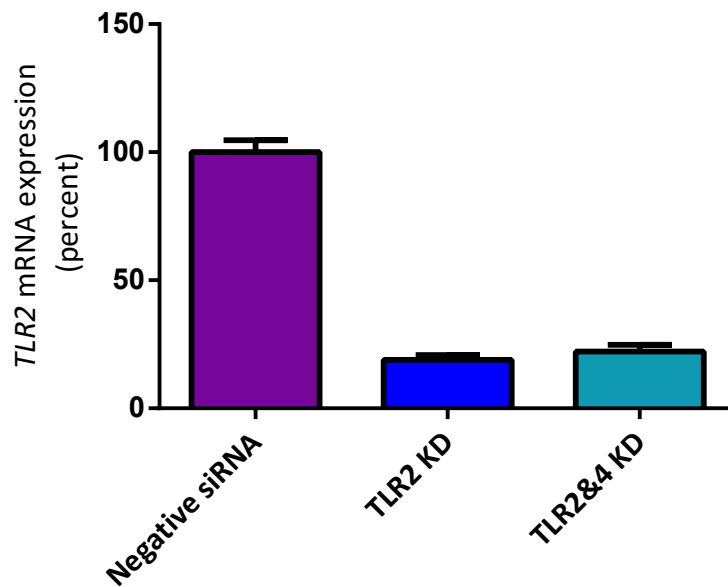

Supplement: Supplementary file 3 [file CTI2-7-e1021-s003.pdf]

## Slide 1
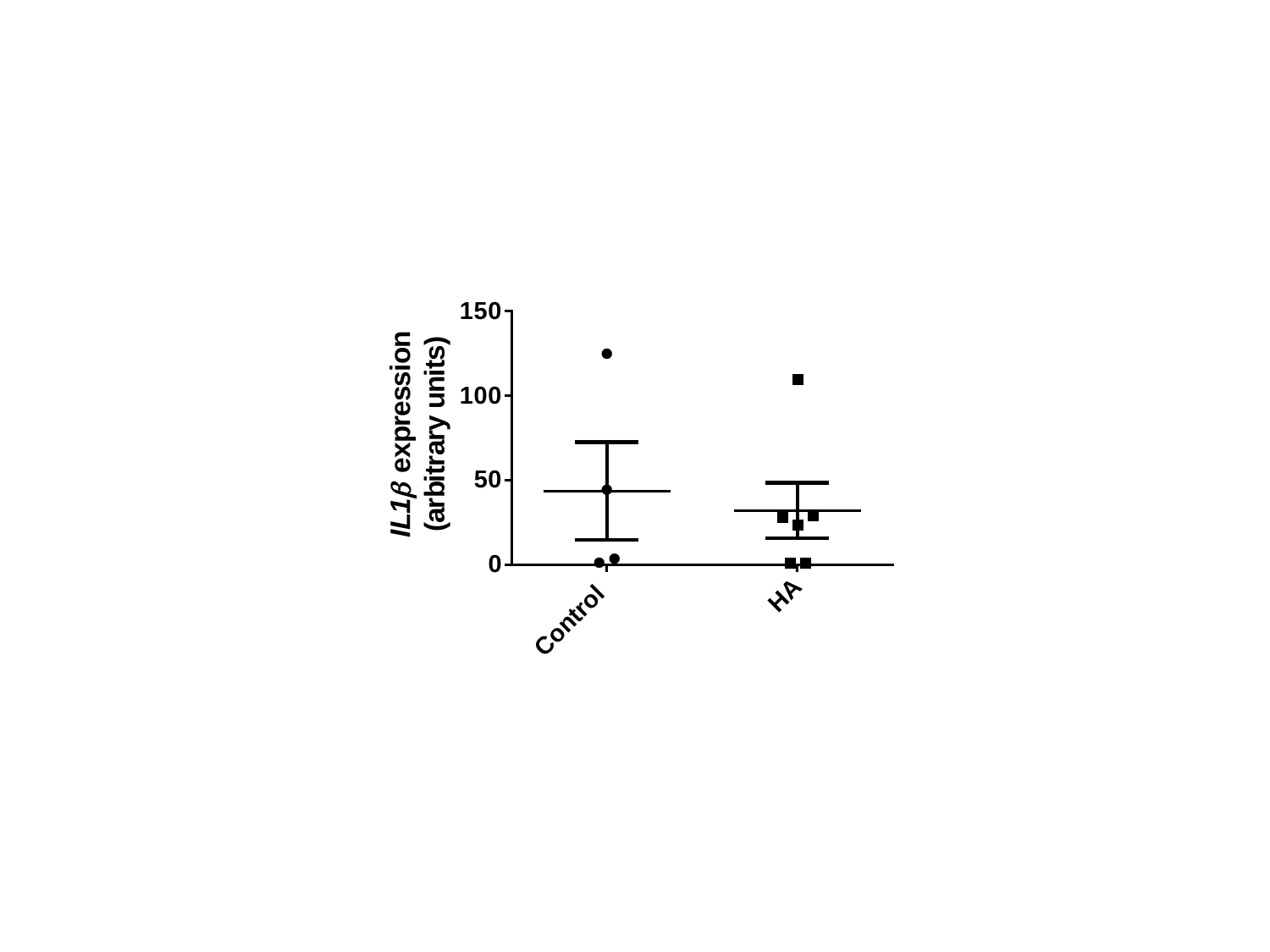

Supplement: Supplementary file 4 [file CTI2-7-e1021-s004.pptx]
