# Supplementary material for: High molecular weight hyaluronic acid: a two‐pronged protectant against infection of the urogenital tract?
Source: Clin Transl Immunology. 2018 Jun 7;7(6):e1021. doi: 10.1002/cti2.1021 (PMC5993165; doi:10.1002/cti2.1021)
Supplement: Supplementary file 5 [file CTI2-7-e1021-s005.pdf]

Gene	Primers		Annealing temperature
	F	R	
DEFB4	CAGCCATCAGCCATGAGGGT	CCACCAAAAACACCTGGAAGAGG	58°C
LCN2	CAAAGACCCGCAAAAGATGT	GGCAACCTGGAACAAAAGTC	58°C
IL8	ATGACTTCCAAGCTGGCCGTGGCT	TCTCAGCCCTCTTCAAAAACTTCT	58°C
IL1b	TGAGCTCGCCAGTGAAATGA	AACACGCAGGACAGGTACAG	58°C
